# Optimizing Contact Network Topological Parameters of Urban Populations Using the Genetic Algorithm

**DOI:** 10.3390/e26080661

**Published:** 2024-08-03

**Authors:** Abimael R. Sergio, Pedro H. T. Schimit

**Affiliations:** Informatics and Knowledge Management Graduate Program, Universidade Nove de Julho, Rua Vergueiro, 235/249, São Paulo 01525-000, Brazil; abimaelr.sergio@uni9.edu.br

**Keywords:** epidemiological modeling, complex networks, genetic algorithms, social networks, parameter estimation

## Abstract

This paper explores the application of complex network models and genetic algorithms in epidemiological modeling. By considering the small-world and Barabási–Albert network models, we aim to replicate the dynamics of disease spread in urban environments. This study emphasizes the importance of accurately mapping individual contacts and social networks to forecast disease progression. Using a genetic algorithm, we estimate the input parameters for network construction, thereby simulating disease transmission within these networks. Our results demonstrate the networks’ resemblance to real social interactions, highlighting their potential in predicting disease spread. This study underscores the significance of complex network models and genetic algorithms in understanding and managing public health crises.

## 1. Introduction

Mathematical epidemiological modeling was a very important tool during the COVID-19 pandemic. Traditional models complemented by more computationally intensive approaches reflected the increasing complexity and demands of modern epidemiological research [1,2]. Moreover, we have never had so many detailed data to be used in different models and approaches [3,4,5]. Despite the innovative character of COVID-19 epidemiological modeling, the objectives of these studies may come from a book from 1991, which are to dissect the mechanics of an epidemic’s spread within a region and to develop effective strategies to mitigate its impact [6].

Advancements in network theory have significantly enriched the modeling of disease transmission, especially when spatial factors are non-negligible. Researchers have turned to the concept of graphs or networks, wherein populations are modeled as nodes (representing individuals) interconnected by edges (symbolizing social and/or spatial contacts) [7,8]. Regular networks, where each node has an identical number of connections, are too simplistic to accurately represent the multifaceted nature of real-world social networks. Thus, complex networks, with their diverse and intricate connection patterns, have become the standard in modeling population dynamics [9,10]. In epidemiological contexts, there are two most commonly used approaches to define a node. In the basic approach, individuals are represented as nodes, and interactions between them are depicted as undirected edges, forming the basis of the population model [7,11]. A node can also be seen as one *patch*, referring to a specific area within a landscape that is different in character from its surroundings. The linking of these patches are the movement rates between patches, configuring a metapopulation, which consists of a group of spatially separated populations that interact at some level. Therefore, *multi-patch* epidemiological models have gained attention in the past decades [12,13,14,15,16]. Therefore, complex networks have become a fundamental tool for modeling population dynamics. Well-established network models have been used, such as small-world and scale-free models [17,18,19], but intricate connection structures [20,21] and networks that incorporates spatial patterns [22,23,24] have also been considered. Since the start of the COVID-19 pandemic, the use of complex networks methods has become crucial in understanding and simulating various aspects of population distribution, interactions, and dynamics. This field of study has included a wide range of models to closely connect individuals and capture the interaction dynamics between susceptible and infected people. Some authors have focused on the movement of populations across transportation networks [25,26], as well as the movement of metapopulations within different patches [16,27,28]. Additionally, multi-patch models that include various types of transportation between nodes have been explored [25,29]. The use of mobile device location data [30] and commuting patterns [31] enabled the creation of a detailed network of individuals movement. Furthermore, the approach of complex networks has been crucial in tracking contact networks to discover the chain of infection [32], as well as in creating groups of individuals to identify infection cases [33], highlighting the versatile use of these methods in pandemic analysis and response planning.

Pujante-Otalora et al.’s systematic review [2] analyzes networks in infectious disease modeling, focusing on 112 studies of network-based transmission. It categorizes networks into contact, relationship, metapopulation, and multilayer types. The study highlights the widespread use of relationship networks, which indicate social connections potentially leading to physical contact, and notes less frequent use of contact and multilayer networks. Metapopulation networks, tracking location-based movements, were also significant. The paper points to an increasing interest in using networks for disease transmission dynamics, underscoring the importance of epidemiological models in quantifying disease spread and assessing intervention impacts. Additionally, the review discussed the variety and granularity of the data sources used to test these models. However, detailed individual movement and contact data were sparingly used in the papers, indicating an area for further detailed research and analysis.

In the set of complex network models, the most used for epidemiological investigations are the *Erdös–Rényi*, *small-world*, *scale-free*, and *Barabási–Albert* frameworks [7,8,9,19]. Each of these models delineates its own methodology for network construction according to specified input parameters. Therefore, considering that individual contacts create a network that can be used as a population model for a disease to spread, this study interrogates whether it is feasible to discern input parameters that can create networks mirroring the dynamics of disease propagation in an urban setting. To address this query, we propose a genetic algorithm—a well-known optimization technique prevalent in epidemiological modeling for parameter estimation. The following sections of this paper will elucidate the efficacy of this approach in parameter determination, thereby contributing to a deeper understanding of disease spread within complex networks.

In the growing complexity of epidemiological models, a number of works have been proposed for parameter estimation of these models [34,35,36,37,38,39,40,41,42,43,44] using algorithms based on deterministic methods (Newton’s method, Gauss–Newton, Levenberg–Marquardt) [45] and statistical methods (maximum likelihood estimators, Bayesian, method of moments, minimum mean square error, principal differential analysis, among others) [46]. Traditional statistical methods, which are based on assumptions such as linearity, independence of observations, and normality of errors, often prove inadequate in the face of the intricate nature of contemporary dynamic systems. These systems, responsible for describing the spread of diseases with increasing detail and specificity, exhibit characteristics such as non-linearity, interdependence among variables, and the presence of latent variables [47]. Intensifying this situation, the growing expansion of parameters to be estimated due to the system’s increasing complexity, coupled with the phenomenon known as the “curse of dimensionality”, brings forth significant computational challenges [48]. For example, the variation of a SIR model (susceptible–infected–recovered model) presented in [49] for diseases with airborne transmission and lifelong immunity included three states and four parameters related to the transition between disease states. In contrast, a similar COVID-19 model that considered hospitalized states used eight states and twelve parameters for the same part of the model. However, these values did not account for parameters related to the population network in either study.

On the other hand, artificial intelligence (AI), and particularly meta-heuristic methods, have been shown to be capable of modeling and estimating non-linear, interdependent, and high-dimensional data, learning directly from the data without the need for prior assumptions about the model [50]. Furthermore, these methods are highly scalable and can handle large volumes of data—a resource that is increasingly valuable in the era of “big data” [51]. Their flexibility and ability to cope with complexity make AI an increasingly popular tool in the modeling and estimation of complex dynamic systems.

The genetic algorithm (GA), as introduced by John Henry Holland, is a probabilistic search technique inspired by Darwin’s principle of natural selection and survival of the fittest. It mimics the genetic and evolutionary mechanisms of species to optimize or minimize a function. The GA operates on the concept that robust solutions are more likely to survive and reproduce, passing on their genetic traits to subsequent generations. This method employs a biological nomenclature, drawing parallels between natural systems and GA elements. Key components of the GA include the fitness function (which evaluates the suitability of solutions), chromosomes (representing potential solutions), selection (picking chromosomes based on fitness for reproduction), crossover (mixing genes of selected chromosomes to create offspring), and mutation (random alterations in new-generation chromosomes) [52,53].

The GA process begins with an initial, randomly generated population of chromosomes. It assesses the fitness of each chromosome, favoring those with a higher fitness for reproduction. This leads to the generation of a new population through the recombination of superior chromosomes. The procedure repeats iteratively until a pre-set termination condition is met, enhancing the characteristics of subsequent generations. The replacement of the entire population follows a specific process involving fitness evaluation, selection of the fittest chromosomes, application of crossover and mutation, and population replacement. The fitness function plays a crucial role in this process, determining which chromosomes will reproduce by assigning a fitness measure to each, thus guiding the selection of the most fit individuals [54,55].

In the field of epidemiological modeling, genetic algorithms have been widely employed for estimating a wide range of parameters. In general, the parameters set to be estimated include the chromosome and are solution candidates. These parameter values are optimized, aiming at a fitness function that can be formulated using a temporal series of real epidemiological variables such as the daily number of cases and deaths [56,57] or the picture of an endemic disease in a population [58], for instance.

One of the primary focuses has been on generic disease rates, including contact, recovery, transmission, and mortality rates. These rates are essential for understanding the dynamics of disease spread in a population [58,59]. Additionally, the GA has been used in estimating the rates related to other disease transition states, such as cured cases, deaths, and different phases within a single state, such as infected asymptomatic and symptomatic individuals and those hospitalized and hospitalized in the intensive care unit, for instance [60,61]. The estimation of rates related to disease control, such as vaccination, quarantine, protection, and treatment [62,63], also highlights the application of the GA in refining the transition state rates of a wide range of compartmental models. Moreover, it underscores the versatility of the GA in handling various aspects of disease management. Lastly, the GA has been used to estimate parameters concerning broader epidemiological factors. These include population structure and movement, as well as initial conditions for simulations, which are vital for creating realistic and applicable models [64,65], thus making it a valuable tool in epidemiological research and public health policy formulation.

A comprehensive overview of studies employing genetic algorithms as an optimization tool for epidemiological parameters is provided in Table 1.

**Table 1:** Summary of studies using genetic algorithm for the optimization of epidemiological parameters.

Variables/Parameters	Papers
Disease rates (contact, recovery, transmission, mortality rates)	[56,57,58,59,60,61,62,63,64,65,66,67,68,69,70,71]
Other rates related to the disease transition states (cure, death, phases of a same state)	[56,57,59,60,61,62,64,65,66,67,71]
Rates related to disease control (vaccination, quarantine, protection, treatment rates, therapies settings)	[56,57,61,62,63,66,72,73]
Period parameters (latency, infectiousness)	[57,59,62,64,65,66]
Parameters of statistical models	[69]
Number of cases, deaths, recovered patients, and susceptible individuals	[64,72,74,75,76]
Population structure, movement, migration	[62,64,65]
Initial conditions for simulations	[57,64,65,67]

In this study, the objective is to identify the optimal topological parameters for the networks, generated through contact interactions within a population, that most accurately align them with the temporal progression of both real and simulated COVID-19 case numbers. Therefore, we employ the GA to optimize the parameters characterizing networks based on the small-world and Barábasi–Albert models. While other network models were also evaluated, these two models yielded the most promising results.

The remainder of this paper is structured as follows: Section 2 details the epidemiological models and the GA methodology employed. The findings are then presented in Section 3, followed by a comprehensive discussion in Section 4.

## 2. Methodology

In this paper, we propose a epidemiological model based on the dynamics of a network formed through individual interactions within a population. This model incorporates elements from two previous models, as detailed in [19,77]. Although it employs the straightforward framework of a SIR model, we demonstrate its effectiveness in identifying the optimal network structure for our study objectives. Following this, we will detail the specific GA operators and the reasoning for their selection.

### 2.1. The Epidemiological Model

In this paper, we model the population as a network G=(V,E) with *N* nodes, where each node represents a place with an individual classified into one of the disease compartments. At the start of each time step, individuals either remain at their current node or move to an adjacent node, with an equal probability for each option. For instance, if a node has five neighbors, the probability of an individual staying at that node is 1/6, and similarly, the probability of moving to any given neighboring node is also 1/6. Once all the individuals have either remained at their nodes or moved to adjacent ones, nodes may contain more than one individual. In these cases, we have a *group*, which is a temporary grouping of individuals where disease transmission occurs. During each time step, susceptible individuals engage in *C* interactions within their group. An interaction could be interpreted as a period during which individuals are in contact. After forming these groups, the individuals return to their original nodes.

Among these interactions, $C_{I}$ is defined as the number of contacts with infected individuals. Consequently, the probability $P_{i}\left(C_{I}\right)$ of a susceptible individual becoming infected is given by $P_{i}\left(C_{I}\right)=1-e^{-kC_{I}}$, where *k* is a parameter related to the infectivity of the disease. Following infection, individuals have a probability $P_{c}$ of recovering or a probability $P_{d}$ of succumbing to disease-related complications. Recovered individuals face a general mortality probability of $P_{n}$. To maintain a constant population size, new susceptible individuals are introduced to replace those who have died.

Each simulation initiates with a set of initial conditions, S(0),I(0), and R(0), and proceeds through time steps that encompass *C* interactions per individual. At the end of each time step, state transitions are calculated and the new states are updated synchronously. This process is repeated for $t_{s}$ time steps. This epidemiological model is similar to the one used on [77], which was based on classical variants of the SIR model used in [49,78,79,80,81,82].

In this paper, we focus on the small-world (SW) and Barábasi–Albert (BA) network models. Comparative evaluations with other network models revealed no significant advantage over these two. The essence of the SW model is rooted in a regular network structure, where *n* nodes are initially positioned in a ring configuration. Each node in this ring is then connected to its closest $m_{sw}$ neighbors. The distinguishing feature of this model arises when some of these edges are rewired with a probability of $p_{sw}$. This rewiring introduces occasional long-range connections, breaking the initial regularity. However, the network still preserves much of its local structure. As a result, we obtain a unique combination of local clustering and a short average path length—characteristics emblematic of a small-world network.

The BA model, proposed by Albert–László Barabási and Réka Albert, hinges on two primary principles: growth and preferential attachment. Starting with a small number of interconnected nodes, the network grows by adding new nodes over time. Each new node connects to *m* existing nodes. The probability of a new node connecting to an existing node *i* among all *j* nodes is given by $\pi_{ba}\left(k_{i}\right)={\left(k_{i}/\sum k_{j}\right)}^{\gamma_{ba}}$, a function of the degree $k_{i}$ of that node. A total of $m_{ba}$ nodes are generated for each vertex. When $\gamma_{ba}=1$, this becomes the linear, standard, preferential attachment of the BA model. This mechanism suggests that nodes with many connections are more likely to receive even more connections, leading to the emergence of hubs with a very high number of links.

These two models capture essential aspects of real-world networks. While the SW model elucidates the “six degrees of separation” phenomenon, indicating that any two nodes in the network can be connected through a surprisingly short path, the BA model sheds light on the ubiquity of hubs in many natural and man-made networks.

To employ iGraph in creating these networks, one would typically input parameters such as the size of the network, $m_{sw}$, $p_{sw}$, $m_{ba}$, and $\gamma_{ba}$. Note that despite the dynamic processes involved in the creation of these networks for both models, we consider the networks that are the final results according to the model creation process, with nodes and edges determined before using the epidemiological process. The library also facilitates extracting the topological properties that are essential for a deep understanding of the network’s structure and function. iGraph is a network analysis package for C, R, Mathematica, and Python [83]. Here, the C version was used.

### 2.2. The GA Model

In this paper, we employ a genetic algorithm optimization procedure to identify suitable input parameters that facilitate the generation of networks replicating the dynamics of COVID-19 within real populations. We have selected several municipalities within the state of São Paulo, Brazil, as our primary case studies. However, it is worth noting that the proposed methodology remains applicable to any urban setting, provided there are comprehensive data detailing the temporal progression of COVID-19 within that locality. Multiple genetic algorithm configurations were meticulously evaluated throughout this research. The culminating parameter selections presented herein are posited as viable candidates, especially for challenges where the objective or fitness function pertains to temporal data series.

Therefore, the chromosome, initial conditions, recombination, mutation, fitness, and other GA properties are described as follows.

The chromosome represents a candidate solution that closely approximates how the real population is connected. We consider a solution as the combination of input parameters used to create the networks ($[m_{sw},p_{sw}$ for the small-world networks, and $m_{ba},\gamma_{ba}$ for the Barabási–Albert networks), as well as the number of interactions per time step, *C*. Each of these parameters is considered to be a gene, and the set of genes, which combined represent a candidate solution, is the chromosome. This is represented by $c_{i}=\left[m_{sw},p_{sw},C\right]$ for small-world networks and $c_{i}=\left[m_{ba},\gamma_{ba},C\right]$ for Barabási–Albert networks.

The number of candidate solutions is denoted as $N_{G}$. At the beginning of the GA process, the parameter values of the candidate solutions are randomly set. Then, the first step of the GA takes place, which is to simulate the epidemiological model in the networks. We now have $N_{G}$ candidate solutions and the epidemiological simulation results for each one of them.

These simulation outputs need to be compared. Therefore, a fitness function is chosen to return a numerical value representing the fitness of the potential solutions, i.e., the simulation outputs closest to the real data. After many tests, the accumulated number of cases was found to be the best data to use in this situation. Consider that t(0),t(1),…,t(k−1) is the time evolution of the real accumulated cases in a city, and s(0),s(1),…,s(k−1) is the time evolution of simulated accumulated cases for the same city, both with *k* days. The fitness function that yielded the best results can be defined as follows:

(1) $$FF\left(s,t\right)=\frac{1}{1+\sqrt{\left(\frac{1}{k}\sum_{i=0}^{k-1}{\left(t\left(i\right)-s\left(i\right)\right)}^{2}\right)+{\left(\sum_{i=0}^{k-1}t\left(i\right)-\sum_{i=0}^{k-1}s\left(i\right)\right)}^{2}}}$$

Once evaluated by the fitness function, i.e., once we have a numerical value for each candidate solution, the formation of the new generation of the GA population takes place. The first process is the recombination of the chromosomes, where $g_{N}$ chromosomes are selected with a probability proportional to their fitnesses, forming $g_{N}/2$ pairs. To match these pairs, the first chromosome is aligned with the second, the third with the fourth, and so on. Each pair swaps their genes with a probability of $g_{c}$ per gene in the crossover process. After this process, we have $g_{N}$ new chromosomes.

For this set of chromosomes, there are two more steps before they form the next generation. A Gaussian mutation is applied to the genes of the chromosomes, with the mean being the current value of the gene and the standard deviation being the $g_{s}$ of the current value of the gene. The last step is elitism, where the $g_{e}$ chromosomes with the highest fitness function values randomly replace the $g_{e}$ chromosomes being processed after the mutation process. Note that $g_{e}$ chromosomes are always copied from one generation to the next. We then have a new generation with $g_{N}$ chromosomes that will be used to simulate the SIR model. This GA process is repeated $g_{t}$ times.

## 3. Results

In this section, we present how the GA performed for some cities in the state of São Paulo, Brazil. Cities with populations of up to 400,000 inhabitants were chosen: Águas de Santa Bárbara, Assis, Atibaia, Avaré, Bauru, Bernardino de Campos, Boituva, Bragança Paulista, Cerqueira César, Embu das Artes, Embu-Guaçu, Itapetininga, Jundiaí, Mogi das Cruzes, Ourinhos, Piraju, Presidente Prudente, and Santa Cruz do Rio Pardo.

By considering one time step of the simulation being one day, and taking into account the initial phases of the COVID-19 pandemic, the epidemiological parameters can be estimated from pertinent academic sources [3,84,85]. Therefore, if the recovery span for those infected stands at 21 days, we have $P_{c}=1/21$. Given the potential for 1% of the infected population to experience severe complications leading to mortality, the value $P_{d}=0.01$ has been assigned to represent this likelihood. With reference to Brazilian statistics indicating an average life expectancy of 78 years, coupled with the presumption of immunity persisting for a duration of four months (or until exposed to a novel variant), the probability $P_{n}$ is determined as $P_{n}=1/\left(78\times 365\right)+1/120\approx 0.00837$.

For optimizing the values of $m_{sw}$, $p_{sw}$, $m_{ba}$, $\gamma_{ba}$, and *C* that better replicate the COVID-19 dynamics in a real city, the GA population is considered to be $g_{N}=50$; the gene swap probability is $g_{c}=0.3$; one unchanged chromosome is taken for the next generation in the elitism process, thus $g_{e}=1$; the initial standard deviation for the Gaussian random number generation is $g_{s}=0.1$ of the current value of the variable; and since the GA process runs for $g_{t}=50$ generations, $g_{s}$ decreases by $g_{s}/g_{t}=0.002$ per generation. As an initial condition, genes $m_{sw}$, $p_{sw}$, $m_{ba}$, $\gamma_{ba}$, and *C* start with a random value in intervals of (0,50), (0,1), (0,50), (1,5), and (1,200), respectively. These values were determined after extensive testing using different configurations. The starting point for these tests was the work presented in Table 1, particularly the study by Monteiro et al., 2020 [58].

A summary of the parameter values used in this process is presented in Table 2.

**Table 2:** Parameters for the SIR and GA models.

Model	Parameter	Description	Value	Reference
SIR	N	Population size	City population	[86]
	$m_{sw}$, $p_{sw}$, $m_{ba}$, $\gamma_{ba}$	Parameters for creating the individual networks	Optimized from GA	-
	C	Interaction parameter	Optimized from GA	-
	$P_{i}$	Probability of infection	Calculated for each individual per time step	-
	$P_{c}$	Probability of cure	1/21	[3,84,85]
	$P_{d}$	Probability of death due to disease	0.01	[3,84,85]
	$P_{n}$	Probability of a recovered individual becoming susceptible	0.00837	[3,84,85,87]
GA	$g_{N}$	Size of GA population	50	Experimental
	$g_{c}$	Gene swap probability in crossover process	0.3	Experimental
	$g_{e}$	Elitism	1	Experimental
	$g_{s}$	Standard deviation for Gaussian random numbers generation	0.1	Experimental
	$g_{t}$	Number of generations	50	Experimental

**Figure 1:**
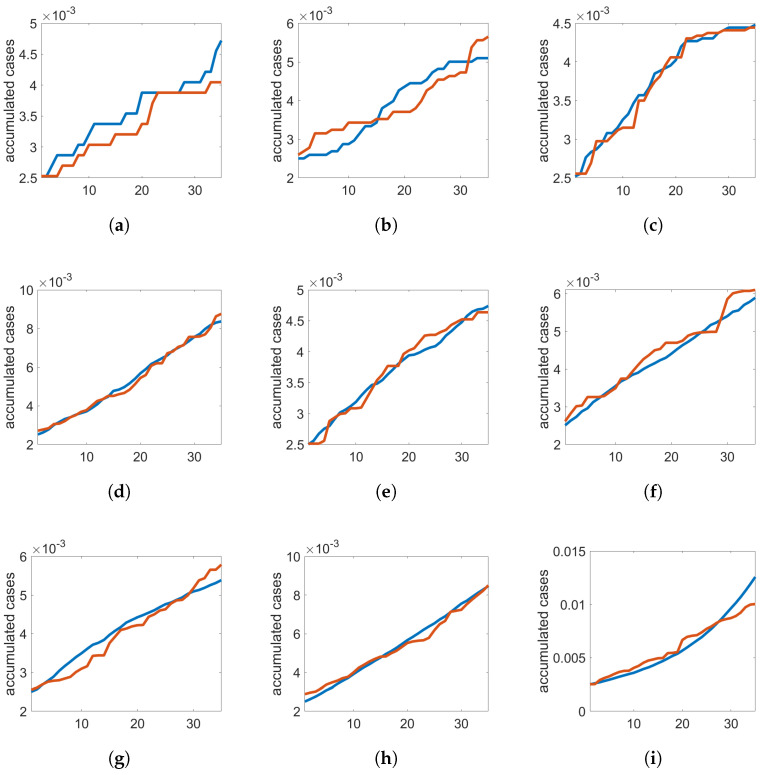
Cumulative evolution of COVID-19 cases over 35 days in selected cities during GA training. Actual data are depicted in red, and simulation results are shown in blue, with each simulation identified by the network model used and the city’s population. (**a**) BA, Águas de Santa Bárbara (5931); (**b**) BA, Bernardino de Campos (10,787); (**c**) BA, Pirajú (28,574); (**d**) SW, Santa Cruz do Rio Pardo (46,110); (**e**) SW, Avaré (87,538); (**f**) BA, Ourinhos (110,489); (**g**) SW, Itapetininga (160,150); (**h**) BA, Presidente Prudente (221,073); (**i**) BA, Jundiaí (407,016).

Therefore, by considering the accumulated number of new cases for the first thirty-five days after this variable achieved 0.25% of a city population, the GA was used to return the topological parameters of the networks that better approximated the real COVID-19 evolution to the simulated data. Figure 1 contains the time evolution of the accumulated number of new cases for nine cities, as well as the population of the cities and the type of network (SW or BA) for the simulation presented. In red, we have the real data for that city, and in blue, we see the results from the simulation. The cities were chosen randomly for representation in the figures. However, the complete data from the GA training are presented in Table 1, considering both networks for all the cities taken into account. In this table, we have the name of the city with its population and the topological parameters found when training with the SW and BA networks. After being trained, each case is run five times to obtain the mean of the error between the real data of the COVID-19 evolution and the simulated data, which are calculated based on the mean absolute percentage error ($e_{MAPE}$).

Note that the results are better to cities with population higher than 10,000 inhabitants, and the error is similar to the SW and BA networks, with a slightly better result for the SW network. However, three cases (Piraju, Santa Cruz do Rio Pardo, and Jundiaí) were better represented by the SW networks, with a significant difference in the value of error $e_{MAPE}$ when compared to the simulation using BA network.

**Figure 2:**
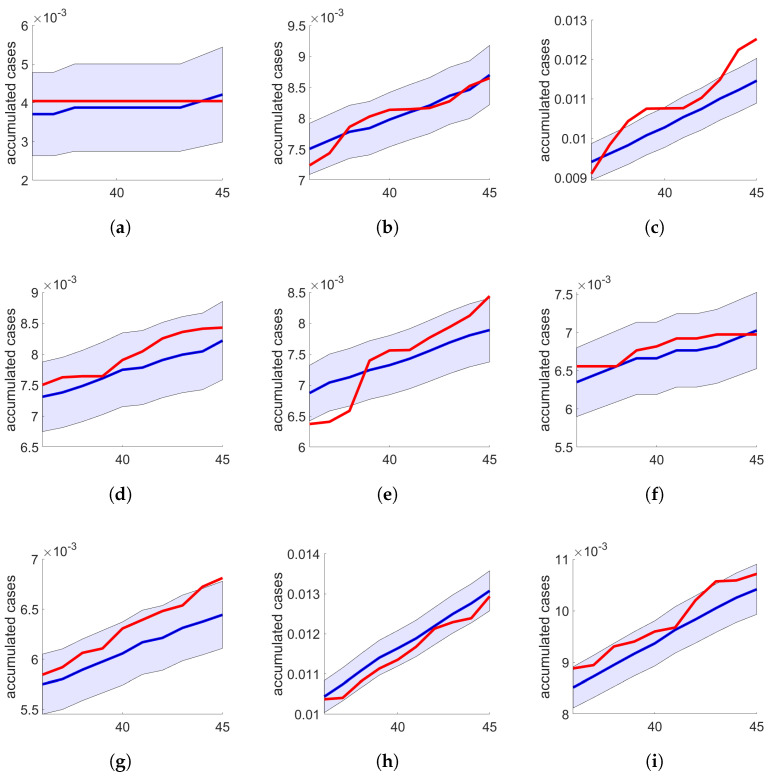
Accumulated COVID-19 case trends over 10 days following the training period in selected cities. Simulations, labeled according to the network model and city population, are compared against actual data: real cases are shown in red and simulated results are shown in blue. (**a**) BA, Águas de Santa Bárbara (5931); (**b**) BA, Pirajú (28,574); (**c**) BA, Bauru (364,225); (**d**) SW, Boituva (57,292); (**e**) SW, Bragança Paulista (163,980); (**f**) BA, Cerqueira César (19,213); (**g**) SW, Itapetininga (160,150); (**h**) BA, Jundiaí (407,016); (**i**) BA, Presidente Prudente (221,073).

Having determined the topological parameters for constructing individual networks in various cities, we next evaluate their effectiveness in predicting disease progression. Figure 2 illustrates the actual versus simulated case evolution over ten days after the initial thirty-five-day training period. The light blue area indicates the training period’s mean absolute percentage error ($e_{MAPE}$). These results suggest the feasibility of utilizing these networks for short-term forecasting beyond the training period. Detailed data from this ten-day post-training simulation are presented in Table 3, where the recalculated $e_{MAPE}$ value for this period is also provided. It is noteworthy that both the SW and BA network models show promising results, although the BA networks exhibited higher error values in certain instances, as seen in the cases of Piraju, Boituva, Avaré, and Jundiaí.

The key outcome of this study is the ability to generate individual contact networks using our model, which can then be applied to analyze disease progression in urban settings. Notably, during the COVID-19 pandemic, these networks experienced significant changes due to factors like lockdowns, social distancing, widespread mask usage, and shifts in behavioral patterns. To adapt to these dynamic conditions, the model can be periodically retrained, thereby refining its capacity to forecast disease evolution more accurately (Table 4).

The final key finding of this study pertains to two specific network properties: the clustering coefficient and network entropy. Briefly, a node’s clustering coefficient represents the proportion of actual connections among its neighboring nodes relative to the total possible connections. The network’s average clustering coefficient is the mean value across all nodes [8]. Typically, in social networks, the global clustering coefficient ($g_{cc}$) exceeds the ratio of the average degree per node ($\bar{k}$) to the total number of nodes (*N*), as indicated by the inequality $g_{cc}>\bar{k}/N$ [10].

Network entropy, derived from information theory, quantifies the heterogeneity of the network’s degree distribution. This study uses the classical Shannon entropy for discrete distributions. The entropy of a node *i* is given by $S_{i}=\ln\left(k_{i}\right)$, with a normalized node entropy of $Hi=\ln\left(k_{i}\right)/\ln\left(N-1\right)$. The network entropy is the average normalized node entropy, $H=\sum {i=1}^{N}\ln\left(k_{i}\right)/\left(N\ln\left(N-1\right)\right)$ [88].

**Figure 3:**
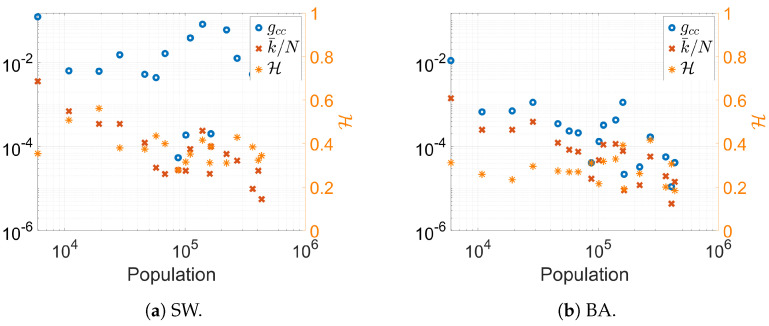
Comparison between clustering coefficient, entropy, and the mean number of edges per node divided by the size of the networks for the data results of both network models considered here.

Figure 3 displays the clustering coefficient, the ratio $\bar{k}/N$, and the network entropy mathcalH as functions of the population size of the cities considered in this study. Interestingly, the inequality $g_{cc}>\bar{k}/N$ holds across all the cities studied. The original study on small-world networks anticipated such outcomes. For Barabási–Albert networks, the difference between $g_{cc}$ and $\bar{k}/N$ is minimal, with both metrics exhibiting lower values compared to small-world networks, as expected [8]. The network entropy values (values on the right y-axis) are slightly higher for small-world networks in smaller cities. For larger cities and Barabási–Albert networks, the entropy ranges from 0.2≤H≤0.4.

Freitas et al. (2019) [88] calculated the clustering coefficient and network entropy for several real networks. For a network of email communications within a university with about 1700 employees [89], the clustering coefficient was $g_{cc}\approx 0.16$, and the entropy was H≈0.25. These values are consistent with those for the smallest city considered here and modeled using the small-world model. Another network considered in their study was a science collaboration network discussed in A.L. Barabási’s book (2016) [90], with a size of N=23,133. This network had a clustering coefficient of $g_{cc}\approx 0.26$ and an entropy of H≈0.16, indicating a high $g_{cc}$ and low H. Given that we are dealing with entire cities, the network parameters extracted are coherent, as we can expect the clustering coefficient to be lower than those of professional networks but higher than $\bar{k}/N$. We can also expect higher network entropy due to the greater diversity within the network.

These results are important, as they demonstrate that networks derived through GA optimization are simplified versions of social networks that are capable of approximately reproducing real infection cases. The methodology outlined in this paper offers a versatile approach for estimating these networks, which can then be used to predict the progression of a disease within a population.

A drawback of the methodology presented here is the computational cost of calculating the clustering coefficient of the networks. A regular personal computer with a 2.8 GHz processor and 16 GB of RAM may require up to four days to complete the task. We simulated the software using appropriate cloud machines with parallel processing, which reduced the processing time to less than two days.

## 4. Discussion

The results obtained in this study offer a framework for dealing with social interaction modeling in the field of mathematical epidemiology. Other network models, topologies, and configurations of social interactions have demonstrated their capabilities for modeling populations and social interactions in studying the dynamics of disease spread, such as multiplex networks [91] and networks with pairwise interactions among individuals [92]. However, the simple complex networks presented here, specifically the small-world and Barabási–Albert models, can be successfully employed to reproduce the dynamics of disease spread within urban populations. Moreover, the use of genetic algorithms for optimizing network parameters has demonstrated not only the feasibility but also the efficiency of this approach in simulating realistic disease propagation scenarios. These findings align with the current literature, emphasizing the need for versatile and adaptive modeling techniques in the face of rapidly evolving public health challenges.

Modeling real networks is challenging due to their complexity and dynamic nature. Researchers have highlighted the difficulties in maintaining the integrity of temporal patterns and accurately reflecting real-world behaviors over time [93,94]. Additionally, capturing the nuances of temporal interactions demands sophisticated methodologies and substantial computational resources [95]. Here, we provided simple models of complex random networks generated through GA optimization that resemble real network patterns for disease spreading dynamics. This similarity is important, as it affirms the model’s potential in forecasting disease evolution in a given population. Additionally, the adaptability of the model in response to changing social behaviors, such as those witnessed during various phases of the COVID-19 pandemic, including lockdowns and social distancing measures, further validates its practical applicability. We also reflect on the broader implications of our research in the context of public health policy and disease management, considering the potential of such models in guiding effective intervention strategies.

Genetic algorithms have effectively optimized a variety of epidemiological models. Typically, these models are calibrated with actual data using GA techniques before their application is broadened to explore different aspects of disease dynamics. As highlighted in the introduction, accurately determining contact, transmission, recovery, and mortality rates is crucial [57,60,61]. This is closely followed by an analysis of other states of disease transition [59,67,71] and rates pertinent to disease control measures [56,66,73]. While the population structure, as well as individual movement and migration, have been considered in previous research, they have not been approached in the manner presented in this paper. In our study, the network shaped by individual interactions plays a significant role in affecting contact and transmission rates. Consequently, the GA method introduced here has precisely fine-tuned the network parameters, demonstrating the method’s efficacy and potential for broader application.

During an actual epidemic, the methodology outlined in this paper can be replicated to assist health authorities in forecasting short-term disease dynamics within a population. By understanding the contact network properties, officials can not only predict how the disease might spread but also simulate various control scenarios to mitigate its impact. These actions might include implementing targeted lockdowns, enforcing strict social distancing measures in identified hotspots, or planning mass testing and vaccination drives in vulnerable areas. Additionally, the model allows for the exploration of more nuanced strategies, such as staggered work hours, temporary closure of high-risk venues, or even predicting the effects of public transportation adjustments on disease transmission. By simulating these interventions, authorities can assess potential outcomes, such as a decrease in infection rates or a flattening of the epidemic curve, and tailor their response plans accordingly. Ultimately, this approach provides a valuable tool for proactive epidemic management, enabling decision-makers to evaluate the efficacy of various control measures before implementing them and to adjust strategies in real-time based on evolving scenarios.

## Figures and Tables

**Table 3:** Results of GA training across network models, detailing optimized parameter values and corresponding mean absolute percentage error ($e_{MAPE}$) during the training period.

City	Population	Network	$m_{\mathrm{sw}}/m_{\mathrm{ba}}$	$p_{\mathrm{sw}}/\gamma_{\mathrm{ba}}$	C	$e_{\mathrm{MAPE}}$
Águas de Santa Bárbara	5931	SW	11.22	0.28	112	0.358
Águas de Santa Bárbara	5931	BA	10.06	1.25	109	0.291
Bernardino de Campos	10,787	SW	56.35	0.74	98	0.098
Bernardino de Campos	10,787	BA	11.36	16.80	51	0.106
Cerqueira César	19,213	SW	127.12	0.55	139	0.088
Cerqueira César	19,213	BA	10.01	9.11	63	0.071
Piraju	28,574	SW	25.48	0.63	96	0.074
Piraju	28,574	BA	17.14	1.42	134	0.130
Santa Cruz do Rio Pardo	46,110	SW	28.66	0.42	172	0.080
Santa Cruz do Rio Pardo	46,110	BA	19.36	5.36	70	0.155
Boituva	57,292	SW	59.70	0.80	161	0.077
Boituva	57,292	BA	19.46	2.30	95	0.089
Embu-Guaçu	68,053	SW	43.05	0.67	144	0.062
Embu-Guaçu	68,053	BA	20.01	4.66	59	0.068
Avaré	87,538	SW	12.39	0.21	98	0.052
Avaré	87,538	BA	34.35	1.93	91	0.138
Assis	101,381	SW	19.78	0.28	158	0.063
Assis	101,381	BA	12.13	7.09	63	0.055
Ourinhos	110,489	SW	30.54	0.44	135	0.056
Ourinhos	110,489	BA	40.83	7.76	52	0.068
Atibaia	139,606	SW	69.46	0.72	164	0.049
Atibaia	139,606	BA	49.78	11.94	62	0.054
Itapetininga	160,150	SW	21.50	0.01	114	0.067
Itapetininga	160,150	BA	109.12	10.22	52	0.052
Bragança Paulista	163,980	SW	52.44	0.79	136	0.054
Bragança Paulista	163,980	BA	10.21	7.90	56	0.065
Presidente Prudente	221,073	SW	23.46	0.33	180	0.047
Presidente Prudente	221,073	BA	25.94	6.15	62	0.078
Embu das Artes	270,790	SW	107.48	0.46	117	0.048
Embu das Artes	270,790	BA	141.91	1.36	128	0.055
Bauru	364,225	SW	69.39	0.02	184	0.055
Bauru	364,225	BA	13.72	9.91	70	0.050
Jundiaí	407,016	SW	33.36	0.48	199	0.038
Jundiaí	407,016	BA	46.42	1.38	148	0.102
Mogi das Cruzes	432,905	SW	44.84	0.49	106	0.049
Mogi das Cruzes	432,905	BA	11.56	12.37	38	0.059

**Table 4:** Outcomes of GA training showing mean absolute percentage error ($e_{MAPE}$) over a ten-day period following training completion.

City	Population	Network	$m_{\mathrm{sw}}$/$m_{\mathrm{ba}}$	$p_{\mathrm{sw}}$/$\gamma_{\mathrm{ba}}$	C	$e_{\mathrm{MAPE}}$
Águas de Santa Bárbara	5931	SW	11.22	0.28	112	0.254
Águas de Santa Bárbara	5931	BA	10.06	1.25	109	0.394
Bernardino de Campos	10,787	SW	56.35	0.74	98	0.290
Bernardino de Campos	10,787	BA	11.36	16.80	51	0.374
Cerqueira César	19,213	SW	127.12	0.55	139	0.090
Cerqueira César	19,213	BA	10.01	9.11	63	0.156
Piraju	28,574	SW	25.48	0.63	96	0.075
Piraju	28,574	BA	17.14	1.42	134	0.876
Santa Cruz do Rio Pardo	46,110	SW	28.66	0.42	172	0.173
Santa Cruz do Rio Pardo	46,110	BA	19.36	5.36	70	0.172
Boituva	57,292	SW	59.70	0.80	161	0.069
Boituva	57,292	BA	19.46	2.30	95	0.295
Embu-Guaçu	68,053	SW	43.05	0.67	144	0.113
Embu-Guaçu	68,053	BA	20.01	4.66	59	0.153
Avaré	87,538	SW	12.39	0.21	98	0.129
Avaré	87,538	BA	34.35	1.93	91	0.377
Assis	101,381	SW	19.78	0.28	158	0.077
Assis	101,381	BA	12.13	7.09	63	0.103
Ourinhos	110,489	SW	30.54	0.44	135	0.146
Ourinhos	110,489	BA	40.83	7.76	52	0.238
Atibaia	139,606	SW	69.46	0.72	164	0.017
Atibaia	139,606	BA	49.78	11.94	62	0.077
Itapetininga	160,150	SW	21.50	0.01	114	0.123
Itapetininga	160,150	BA	109.12	10.22	52	0.098
Bragança Paulista	163,980	SW	52.44	0.79	136	0.118
Bragança Paulista	163,980	BA	10.21	7.90	56	0.084
Presidente Prudente	221,073	SW	23.46	0.33	180	0.039
Presidente Prudente	221,073	BA	25.94	6.15	62	0.167
Embu das Artes	270,790	SW	107.48	0.46	117	0.125
Embu das Artes	270,790	BA	141.91	1.36	128	0.213
Bauru	364,225	SW	69.39	0.02	184	0.112
Bauru	364,225	BA	13.72	9.91	70	0.109
Jundiaí	407,016	SW	33.36	0.48	199	0.025
Jundiaí	407,016	BA	46.42	1.38	148	0.461
Mogi das Cruzes	432,905	SW	44.84	0.49	106	0.157
Mogi das Cruzes	432,905	BA	11.56	12.37	38	0.227

## Data Availability

Data supporting the findings of this study are available upon request made via email.
